# Assessment of risk factors of treatment discontinuation among patients on paliperidone palmitate and risperidone microspheres in france, germany and belgium – a retrospective database study

**DOI:** 10.1192/j.eurpsy.2021.437

**Published:** 2021-08-13

**Authors:** R. Cai, F. Decuypere, P. Chevalier, A. Wimmer, P. Guillon, S. Pype, A. Godet, V. Timtschenko

**Affiliations:** 1 Real World Evidence, IQVIA, Zaventem, Belgium; 2 Analytics Solutions, IQVIA, Zaventem, Belgium; 3 Medical Affairs, Janssen-Cilag, Neuss, Germany; 4 Health Economics, Janssen-Cilag, Issy-les-Moulineaux, France; 5 Medical Advisor, Janssen-Cilag, Beerse, Belgium

**Keywords:** antipsychotics, PP1M, PP3M, risperidone microsphere, treatment continuation

## Abstract

**Introduction:**

Long-acting antipsychotics (e.g. 1-monthly (PP1M) / 3-monthly (PP3M) injection forms of paliperidone palmitate) have been developed to improve treatment continuation in schizophrenia patients.

**Objectives:**

To assess risk factors of treatment discontinuation in patients on paliperidone palmitate and risperidone microsphere. Additionally, treatment continuation between patients with PP1M and PP3M was compared.

**Methods:**

The IQVIA Longitudinal Prescription databases were used. Risk factors of treatment discontinuation were identified by a multilevel survival regression using Cox proportional hazards model. Kaplan Meier analyses were performed by identified significant risk factors.

**Results:**

25,361 patients (France: 9,720; Germany: 14,461; Belgium: 1,180) were included. Over a one-year follow-up period, a significant higher treatment continuation was observed for patients newly initiated on paliperidone palmitate (46.2%) than those initiated on risperidone microspheres (14.6%). Additionally, a significantly higher treatment continuation was found for ‘stable’ PP3M patients (81.8%) than ‘stable’ PP1M patients (62.9%). Patients were more likely to discontinue when drugs prescribed by GP only (HR = 1.68, p < 0.001 vs. psychiatrist only) or being females (HR = 1.07, p < 0.001), whereas discontinuation rate decreased with age (31-50 years: HR = 0.95, p = 0.006 and > 50 years: HR = 0.91, p < 0.001 vs. 18-30 years).

**Conclusions:**

Paliperidone palmitate was associated with a significantly higher treatment continuation than risperidone microspheres. Treatment continuation is likely to be improved by targeting young patients (18-30 years), empowering GPs with mental health knowledge and managing patients by a collaborative primary care-mental health model. Further research is needed to understand why females have more treatment discontinuation.

**Disclosure:**

Rui Cai, Flore Decuypere and Pierre Chevalier are IQVIA employees and served as paid consultants to Janssen during the conduct of this study. Antonie Wimmer, Pascal Guillon, Stefan Pype, Annabelle Godet, Valeria Timtschenko are Janssen employees.

